# *Rumex* Species: Phytochemistry, Pharmacology and Nutritional Potential for Food and Health Applications

**DOI:** 10.1002/fsn3.71300

**Published:** 2025-12-18

**Authors:** Mai Mohamed Gohar, Shahira Mohamed Ezzat, Balakyz Yeskaliyeva, Seham Salaheldin Elhawary, Farid Noshey Kirollos, Aya Khouchlaa, Abdelhakim Bouyahya, Daniela Calina, Javad Sharifi‐Rad, William N. Setzer, Miquel Martorell

**Affiliations:** ^1^ Department of Pharmacognosy, Faculty of Pharmacy October University for Modern Sciences and Arts (MSA) Giza Egypt; ^2^ Department of Pharmacognosy, Faculty of Pharmacy Cairo University Cairo Egypt; ^3^ Faculty of Chemistry and Chemical Technology Al‐Farabi Kazakh National University Almaty Kazakhstan; ^4^ Laboratory of Microbial Biotechnology and Bioactives Molecules, Faculty of Science and Technology Sidi Mohamed Ben ABdellah University Fez Morocco; ^5^ Laboratory of Human Pathologies Biology, Department of Biology, Faculty of Sciences Mohammed V University Rabat Morocco; ^6^ Department of Clinical Pharmacy University of Medicine and Pharmacy of Craiova Craiova Romania; ^7^ Universidad Espíritu Santo Samborondón Ecuador; ^8^ Department of Medicine, College of Medicine Korea University Seoul Republic of Korea; ^9^ Centro de Estudios Tecnológicos y Universitarios del Golfo Veracruz Mexico; ^10^ Aromatic Plant Research Center Lehi Utah USA; ^11^ Department of Chemistry University of Alabama in Huntsville Huntsville Alabama USA; ^12^ Department of Nutrition and Dietetics, Faculty of Pharmacy, and Centre for Healthy Living University of Concepción Concepción Chile

**Keywords:** anticancer, antimicrobial, antioxidant, bioavailability, ethnopharmacology, pharmacological activities, phytochemistry, *Rumex* species, therapeutic potential, traditional medicine

## Abstract

The genus Rumex (Polygonaceae), comprising about 193 species, is widely distributed across temperate and subtropical regions of Europe, Asia, Africa, and North America, and has long been used in traditional medicine worldwide. These species are rich in diverse phytochemicals, including anthraquinones, flavonoids, tannins, naphthalenes, and stilbenes, which contribute to a broad range of biological activities. This review provides an updated synthesis of current knowledge on the taxonomy, phytochemistry, and pharmacological properties of *Rumex* species. Particular attention is given to *R. dentatus* , *R. vesicarius* and *R. crispus* , which exhibit antioxidant, anti‐inflammatory, antimicrobial, hepatoprotective, antidiabetic, and anticancer effects in both in vitro and in vivo studies. Clinical and toxicological aspects, including oxalate accumulation and anthraquinone‐associated adverse effects, are also discussed. Major research limitations include the lack of standardized extracts, insufficient clinical evidence, and poor bioavailability of key compounds. Enhancing bioavailability is vital because many bioactive compounds in *Rumex* are poorly absorbed and rapidly metabolized, which restricts their therapeutic potential. Environmental factors and phenological stages influencing phytochemical expression are highlighted as additional underexplored determinants of bioactivity. By integrating ethnopharmacological knowledge with experimental data, this review identifies future research priorities, including the optimization of formulation strategies, pharmacokinetic evaluation, and clinical validation. Collectively, these efforts may support the development of safe and effective *Rumex*‐based nutraceuticals and therapeutic products.

## Graphical abstract

*Rumex dentatus* , *R. vesicarius* , and *Emex spinosa* are traditionally used for managing gastrointestinal, respiratory, hepatic, and inflammatory disorders. In addition to their ethnomedicinal value, some species are consumed as wild edibles. Pharmacological studies support their antimicrobial, antioxidant, hepatoprotective, cytotoxic, and antidiabetic activities. These effects are linked to bioactive phytochemicals, including anthraquinones, flavonoids, phenolic acids, and fatty acids, underscoring their potential as functional food sources and candidates for evidence‐based phytotherapeutics.

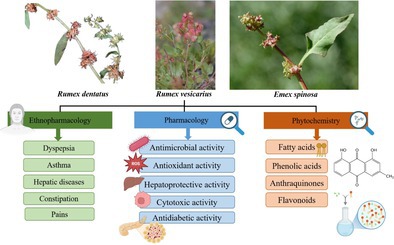

**Abbreviations**
ALP: alkaline phosphatase
ALT: alanine aminotransferase
AST: aspartate aminotransferase
Bcl‐2: B‐cell lymphoma 2
COX: cyclooxygenase
ERK: extracellular signal‐regulated kinase
IC₅₀: half‐maximal inhibitory concentration
IL: interleukin
JNK: c‐Jun N‐terminal kinase
MAPK: mitogen‐activated protein kinase
mTOR: mechanistic target of rapamycin
NFATc1: nuclear factor of activated T‐cells 1
NF‐κB: nuclear factor kappa‐light‐chain‐enhancer of activated B cells
NO: nitric oxide
PI3K/Akt: phosphoinositide 3‐kinase/Protein kinase B (Akt)
PPAR: peroxisome proliferator‐activated receptor
ROS: reactive oxygen species
TNF‐α: tumor necrosis factor‐alpha

## 1 Introduction

The family Polygonaceae, known as the Buckwheat family, comprises approximately 61 genera and over 1100 species worldwide, divided into two subfamilies: Polygonoideae, characterized by the presence of an ocrea, and Eriogonoideae, distinguished by its absence (Borsch et al. 2020; Mabberley 1997). In the Egyptian flora, the family Polygonaceae is represented by eight genera and 26 species (El Gazzar et al. 2023). Globally, members of the Polygonaceae family are widespread, with the greatest diversity found in temperate regions of the Northern Hemisphere, particularly in Asia and North America, but also represented in tropical and subtropical regions worldwide (Gillani et al. 2024). The family consists mainly of herbs, shrubs, and small trees, characterized by simple, alternate leaves and a sheathing stipule known as the ocrea, usually found at swollen stem nodes. Inflorescences bear flowers with 4–6 petaloid tepals, 5–9 stamens, and 2–3 carpels in a superior ovary, producing a 3‐angled achene fruit (Ann and Kron 2003). *Rumex* L., the second largest genus in the family Polygonaceae, comprises approximately 200 species distributed worldwide, with the greatest diversity in temperate regions of the Northern Hemisphere but also represented in tropical and subtropical areas (Jiang et al. 2007). In the Northern Hemisphere, *Rumex* typically flowers between April and May, with seeds ripening from May to June. However, flowering and fruiting periods vary in the Southern Hemisphere (e.g., South America, southern Africa, Australia). Plants belonging to this genus are generally characterized as perennial herbs. Plants belonging to this genus are generally characterized as perennial herbs (Newman et al. 2000). *Rumex* species have had a valued place in global folk medicine, and different species present various biologically active compounds that explain their use in medicine and the pharmaceutical industry (Hasan et al. 1995). To date, 268 phytoconstituents have been identified from 29 *Rumex* species (Li et al. 2022), including anthraquinones, flavonoids, tannins, stilbenes, naphthalenes, diterpene alkaloids, terpenes, and lignans, which exhibit a broad spectrum of biological activities such as anti‐inflammatory, antioxidant, antibacterial, antitumor, and antidiabetic effects (Chelly et al. 2020; Elsayed et al. 2020; Farooq et al. 2020; Froldi et al. 2020; Quradha et al. 2019). *Rumex vesicarius* L., known as “Bladder Dock,” is locally called “Hammad” in parts of Egypt and the Middle East, and is an annual herb (Rahman et al. 2004). It is widely distributed throughout desert and semi‐desert areas of North Africa, Australia, and Asia (Yahya 1990). Phytochemical researchers showed that *R. vesicarius* presents numerous bioactive compounds such as quinones, carotenoids, flavonoids, vitamins (especially vitamin C), lipids, carbohydrates, anthraquinones particularly in roots (emodin and chrysophanol), saponins, tannins, phenols, reducing sugars, triterpenoids, and organic acids. *R. vesicarius* contains several minerals, such as K, Na, Ca, Mg, Fe, Mn, and Cu (Khan et al. 2014). This plant is used in traditional folk medicine to treat asthma, bronchitis, dyspepsia, vomiting, piles, hepatic diseases, constipation, bad digestion, scabies, leucoderma, toothache, appetizer, spleen disorders, pains, heart troubles, flatulence, and as a laxative, stomachic, tonic, diuretic, and analgesic (Batanouny et al. 1999). Several researchers indicated the potential activity of *R. vesicarius* as antioxidant, membrane stabilizing, hepatoprotective, and antifibrotic effects against CCl_4_ intoxication in rats (El‐Hawary et al. 2011). *Rumex dentatus* L., also known as “Dentate Dock”, “Indian Dock”, and “Toothed Dock” (Fatima et al. 2009) is traditionally employed for its bactericidal (Yildirim et al. 2001), anti‐inflammatory (Humeera et al. 2013; Vasas et al. 2015), anti‐dermatitis, astringent, antitumor, diuretic, laxative, cholagogue, and tonic (Litvinenko and Muzychkina 2003). Chemical investigations have shown the presence of anthraquinones in its roots (Liu et al. 1997; Zhu et al. 2006), and various phenolic compounds, flavonoids, and tannins in its aerial parts (Hussain et al. 2010; Zhu et al. 2006). This review provides a comprehensive and updated overview of the botanical characteristics, traditional uses, phytochemical composition, pharmacological properties, clinical relevance, and toxicological data of *Rumex* species, with particular emphasis on mechanistic insights and research gaps that merit further investigation.

## 2 Methodology

**FIGURE 1:**
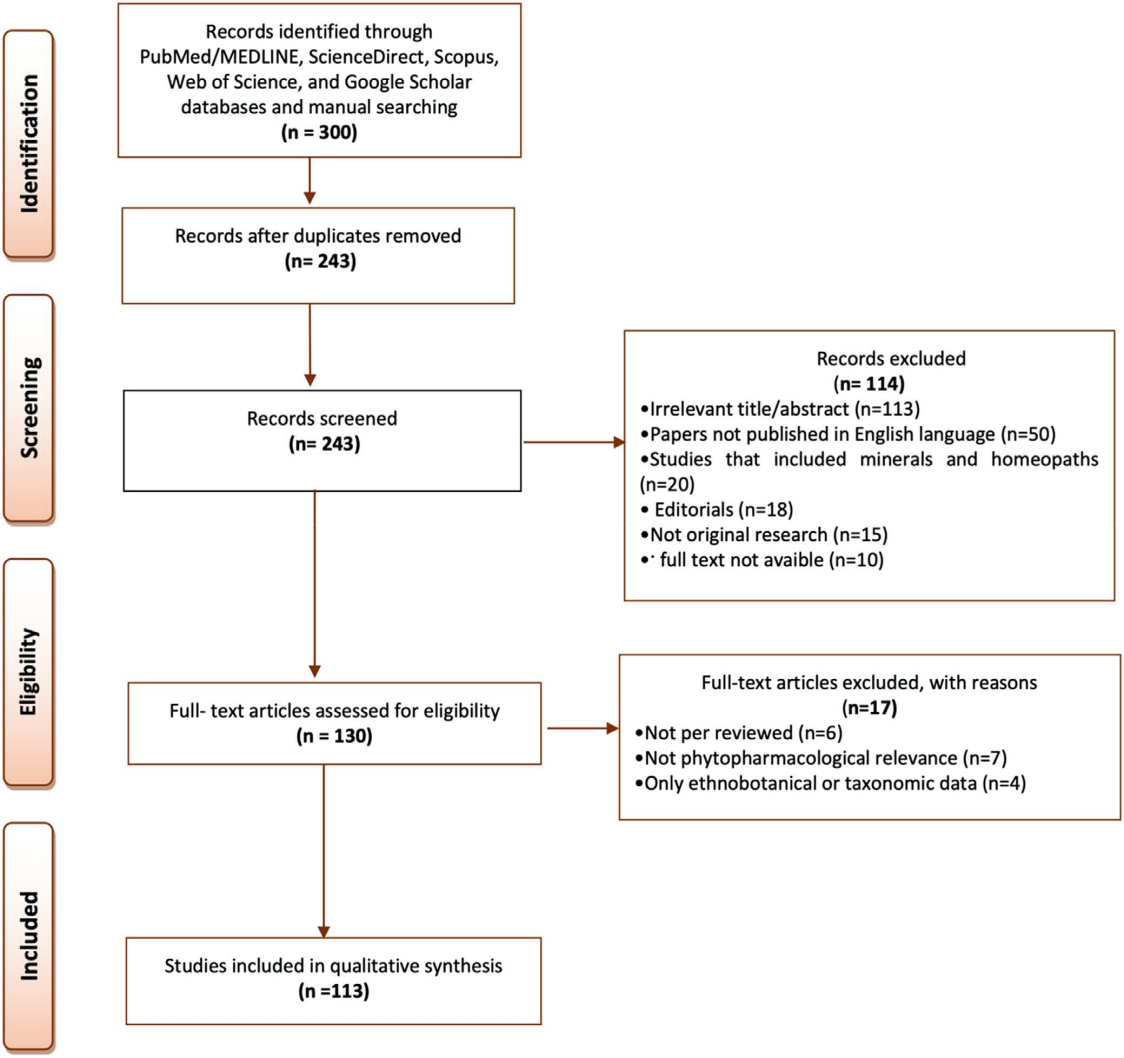
Summary of search and inclusion criteria used in this review.

To conduct this review, a comprehensive and systematic search was performed across several scientific databases, including PubMed/MEDLINE, ScienceDirect, Scopus, Web of Science, and Google Scholar. Search terms were derived from MeSH (Medical Subject Headings) and relevant literature, and included the following keywords: *Rumex*, *Rumex classification*, *Rumex chemistry*, *Rumex species*, *flavonoids*, *anthraquinones*, *plant extracts pharmacology*, *medicinal plants*, *phytotherapy*, *phytochemicals*, *ethnopharmacology*, *bioavailability*, *toxicology*, *antioxidants*, *antimicrobial agents*, *anti‐inflammatory agents*, *antineoplastic agents*, *phytogenic*, *hepatoprotective agents*, and *hypoglycemic agents*, *plant‐derived*. The selection process prioritized studies that provided relevant data on botanical features, phytochemistry, traditional uses, pharmacological properties, and toxicology of *Rumex* species. Inclusion criteria comprised English‐language publications that presented original research on the chemical composition, bioactivity, clinical relevance, or traditional medicinal application of *Rumex* species. Studies were excluded if they were not peer‐reviewed, lacked pharmacological focus, included homeopathic formulations or minerals, or were case reports, editorials, or reviews. Duplicate records were eliminated using citation management tools, and plant taxonomy was validated using the World Flora Online database. Chemical structures were verified through PubChem. Full‐text assessments were performed to confirm eligibility, and only studies meeting the predefined criteria were included in the qualitative synthesis (Figure 1).

## 3 Taxonomy and Botanical Features

**FIGURE 2:**
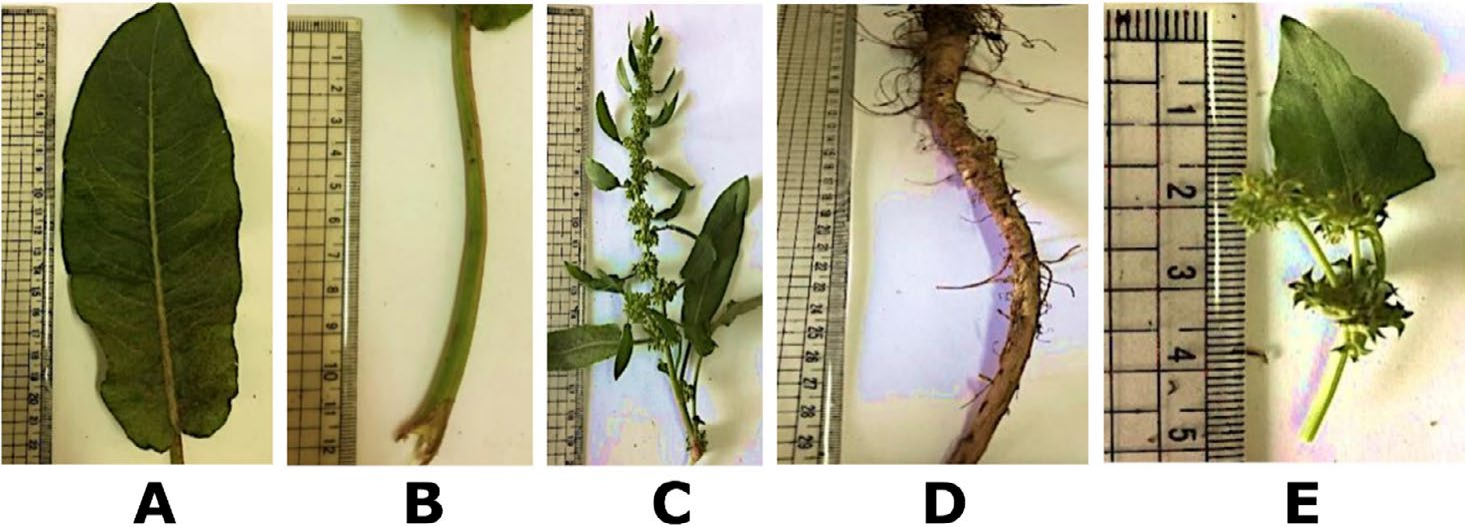
Photographs of *Rumex dentatus* L. (A) leaf, (B) petiole, (C) inflorescences, (D) root, and (E) upper leaves.

Within the family Polygonaceae, which is divided into subfamilies Polygonoideae and Eriogonoideae, the genus *Rumex* comprises approximately 200 species that are broadly distributed across the globe, with its greatest diversity in temperate regions. As described by Sanchez and Kron (2008), Eriogonoideae is defined by the presence of involucres (clusters of bracts). The flowers are enclosed, except in the case of *Gilmania*. This subfamily is distinguished by non‐swollen nodes and the lack of ocrea, although perennial species of *Chorizanthe* in South America are an exception. Some authors reported four tribes in Polygonoideae named Persicarieae, Rumiceae, Polygoneae, and Muehlenbeckia. Emex Neck. ex Campderá, Oxyria, Hill, Rheum L., and Rumex L., is characterized by a fimbriate or pennicilate stigma (Sanchez et al. 2011) (Figure 2).

### 3.1 Polygonaceae Juss

In Egyptian flora, there are 28 wild species in 8 genera from Polygonaceae including herbs, lianas, or shrubs, and rarely trees. In comparison, the Flora of North America recognizes approximately 35 genera and 442 species of Polygonaceae, while the Flora of China reports 13 genera (including two endemic) and 238 species (65 endemic) within China (Boulos 1999 & 2005). Stems are occasionally twining or climbing, typically jointed with nodes that are swollen. Leaves are simple and alternate, though they are rarely opposite or whorled. They can be entire or exhibit various lobed or toothed margins. Stipules are fused to form a membranous sheath known as ocrea. The inflorescence can take the form of heads, spikes, or panicles, consisting of cymose clusters, though it may also feature solitary flowers. Bracts, numbering 1–2, are fused into a sheath called ochreole. Pedicels are generally articulate. The flowers of *Rumex* are actinomorphic, hermaphroditic, or occasionally unisexual, with plants being monoecious or dioecious. The perianth consists of two whorls of sepaloid or colored tepals, numbering 3–6, which may be free or partially fused and are typically persistent. In some cases, the inner whorl becomes accrescent or modified in fruit. The stamens, usually 6–9, sometimes more or fewer, are opposite the tepals and contain 2‐celled anthers. The ovary is superior, 1‐celled and single‐ovuled, with 2–4 styles that are free or partially fused at the base. The fruit is a flat, angular, or winged achene, often enclosed by a membranous or leathery perianth. The endosperm is abundant and mealy, although the embryo is lateral or eccentric, either curved or straight (Boulos 1999 & 2005; Zohary and Feinbrun‐Dothan 1966).

### 3.2 *Rumex dentatus* L.

Common names of *Rumex dentatus* include Devil's thorn, Lesser Jack, Spiny Threecornerjack, and Toothed Dock. In Arabic, it is known by several names, including Dirs al‐Ajooz, Hommeid, Hommal, Khilla, Khella, and Khellala (Takhol 1974). This species is annual, glabrous, with 15–120 cm long or more (Figure 2C). Stems as shown in Figure 2C are erect, either simple or slightly branched. Branches are acute‐divaricated to erect. Basal leaves measure 8–12 cm long, 3 cm broad, ovate‐oblong or oblong‐lanceolate as presented in Figure 2A (Zohary and Feinbrun‐Dothan 1966). Basal leaves are occasionally fiddle‐shaped, with a more or less rounded apex and a base that ranges from truncate to subcordate, often appearing slightly wavy. The petiole is either equal in length to or shorter than the blade. As depicted in Figure 2B, the upper leaves are smaller, short‐petioled, and lanceolate to oblong or elliptical, somewhat acute, minutely crisped, or nearly smooth. The panicles are either branched or nearly simple, consisting of many‐whorled clusters. These whorls are densely flowered, spaced apart, and sometimes merge in the upper section, with nearly all subtended by linear‐lanceolate leaves. The pedicels, jointed near the base, are reflexed and nearly equal in length to the fruiting perianth. Valves measure 3–6 mm in length and 2–7 mm in width, including the teeth. They are 1.5–3 mm long warts, ovate‐lanceolate in shape, somewhat acute, and reticulate, often exhibiting sibilate teeth and occasionally subentire margins. The achene is approximately 2 mm long, trigonous, broadly ovoid, and dark brown (Boulos 1999 & 2005; Zohary and Feinbrun‐Dothan 1966). *R. dentatus* flowering commences in March and finishes in June. *R. dentatus* inhabits the banks of rivers and canals, moist waste ground, and roadsides. *R. dentatus* is widely distributed in the Mediterranean region, Euro‐Siberian, Sino‐Japanese, and also in some tropical regions. In Egypt, this species was recorded in Nile, Oasis, Sinai, and Mediterranean phyto‐geographical regions.

### 3.3 *Rumex vesicarius* L.

**FIGURE 3:**
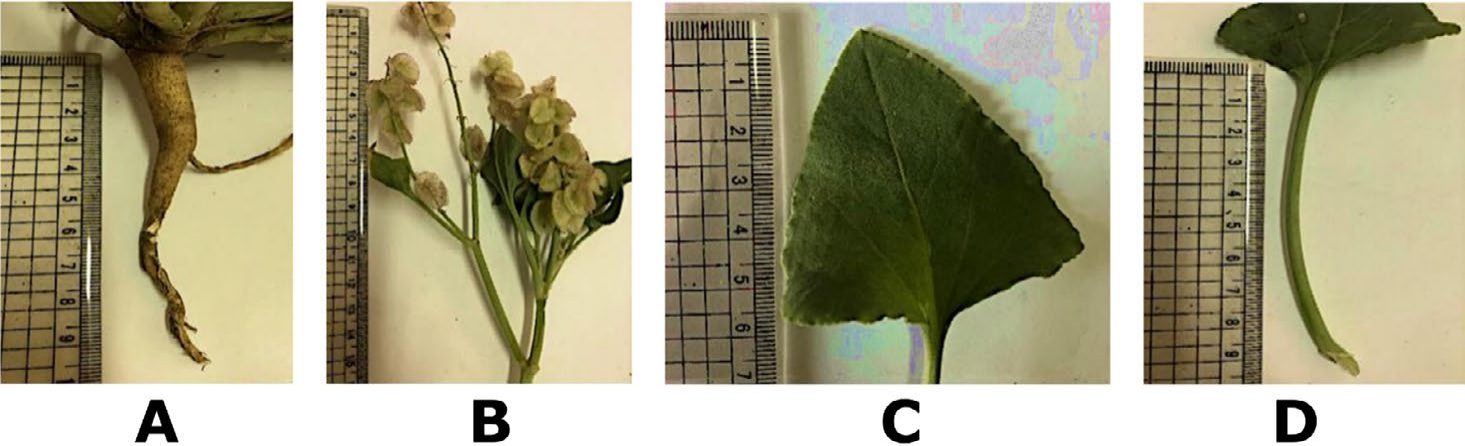
Photographs of *Rumex vesicarius* L. (A) root, (B) inflorescences, (C) leaf, and (D) upper leaf.

The common name of *R. vesicarius* is Ruby dock, and Bladder dock. This plant is known in Arabic names as Hambeit, Hanbeit, Hamaad, Hammaad, Hammaad el‐‘eshb, Hommaad (الحُمَّاض) and Hommeid (الحُمَّيْض) (Takhol 1974). *R. vesicarius* is an annual plant, green‐glaucescent, glabrous, 10–60 cm long or more as in Figure 3B. Stems as shown in Figure 3B are decumbent ascending, branching from the base rather thick. Leaves as shown in Figure 3C measured 7 cm long, and 1.5–5 cm broad, are fleshy, petiolate, ovate to deltoid or oblong‐triangular, cuneate or truncate, subcordate or sub hastate at the base, more or less obtuse, entire. Flowers as shown in Figure 3B are hermaphrodite or unisexual, racemose, or paniculate. Pedicles are single in axils, often bearing 2 flowers as in Figure 3B, jointed below the middle, elongated, and reflexed in fruit. Valves measured 1–2 cm long and are longer than pedicles. There are membranous, subequal, suborbicular, narrowly sinuate‐cordate at the base and apex, longitudinally folded and hence more or less ovate in outline, concealing the second flower, entire, purplish, and netted‐veined, without marginal vein, 2 of the 3 valves bearing a basal wart. Achene measured 3 mm long or longer, are trigonous, ovoid, acuminate, and brownish; those of the first flowers are somewhat longer than those of the second flowers (Boulos 1999 & 2005; Zohary and Feinbrun‐Dothan 1966). *R. vesicarius* flowering commences in February and finishes in May. *R. vesicarius* habitats are desert roads, rocky, and sandy desert wadis. *R. vesicarius* is widely distributed in North Africa, Southwest Asia (extending to South Greece), eastwards to North India and Afghanistan. In Egypt, the species was recorded in the Mediterranean, Desert, Sinai, Red Sea, and Gebel‐Elba phyto‐geographical regions.

## 4 Phytochemistry

Several researchers have been attracted to study the chemical composition of many species belonging to the genus *Rumex* because of their medicinal properties. Numerous classes of phytocompounds appearing in different species belong to the genus *Rumex* (Shafiq et al. 2017). Methanolic extract of *Rumex dentatus* has shown the presence of saponins, alkaloids, tannins, and anthraquinones although flavonoids were found in both methanolic and hexane extracts (Fatima et al. 2009). Most of the species under this genus, such as *Rumex japonicus* Houtt., *Rumex patientia* L., *Rumex crispus* L., *Rumex hymenosepalus* Torr., and *Rumex dentatus* contain flavonoids, anthraquinones, and triterpenoids (Rao et al. 2011). Moreover, flavonoids were abundant in *R. vesicarius* although *R. dentatus* was characterized by the abundance of anthraquinones and chromones (Elfotoh et al. 2013). The phytochemical contents of Rumex species can vary significantly depending on the harvest season, geographical location, soil composition and other environmental conditions, which may influence their pharmacological activity and therapeutic consistency.

### 4.1 Fatty Acids

Several fatty acids have been identified and isolated from different species of the genus *Rumex*.

**FIGURE 4:**
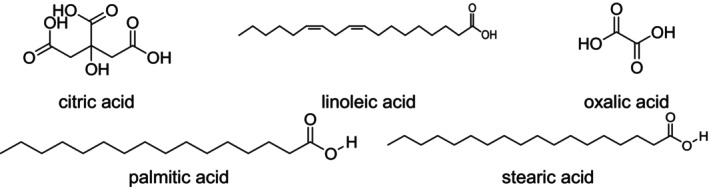
Structures of the reported acids and fatty acids: Short‐chain organic acids such as oxalic, citric, and tartaric acids contain multiple carboxyl groups and exhibit strong metal‐chelating and acidifying properties, contributing to antimicrobial effects. In contrast, long‐chain saturated fatty acids such as palmitic and stearic acids primarily support membrane structure and have limited direct bioactivity. Among unsaturated fatty acids, linoleic acid—characterized by two double bonds—has demonstrated antioxidant and anti‐inflammatory properties due to its unsaturation and role in lipid signaling.

Therefore, one important group of fatty acids includes two types: essential fatty acids, such as linoleic acid, and non‐essential fatty acids, such as saturated acids like palmitic acid and stearic acid, as shown in Figure 4. *Rumex induratus* Boiss. & Reut. (syn. *Rumex scutatus* subsp. *induratus* (Boiss. & Reut.) Malag.) has been reported to contain fatty acids such as linoleic acid, palmitic acid, and stearic acid. In contrast, *Rumex abyssinicus* Jacq. has been found to contain high levels of oxalic acid, a naturally occurring organic acid known for its antinutritional properties Figure 4. Moreover, *Rumex nervosus* Vahl was reported to contain organic acids such as citric acid and tartaric acid. The crude extracts of these plants exhibited antibacterial activity, which has been attributed to the presence of oxalic acid, citric acid, and tartaric acid (Shafiq et al. 2017). In addition, volatile compounds derived from fatty acid metabolism, including 4‐hexen‐1‐ol and 2,4‐hexadiene‐1‐ol, were detected in R. induratus (Mabberley 1997). Furthermore, *R. dentatus* and *R. vesicarius* have been reported to contain high levels of fatty acids such as palmitic, linoleic, and oleic acids (Fatima et al. 2009).

### 4.2 Phenolic Acids

**FIGURE 5:**
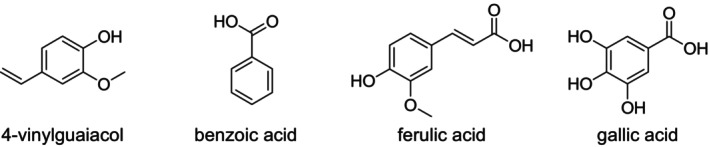
Structures of the reported phenolic acids. Hydroxyl groups on the aromatic ring enhance antioxidant activity, with gallic acid (three–OH) being the most potent. Ferulic acid's conjugated side chain improves radical stabilization. 4‐vinylguaiacol's vinyl group contributes to moderate antioxidant and antimicrobial effects. Benzoic acid, with no additional –OH groups, shows weaker antioxidant activity but retains antimicrobial activity.

Several phenolic acids have been isolated from *Rumex* species. As shown in Figure 5, benzoic acid has been identified in *R. induratus*, occurring naturally in free or esterified forms such as methyl or ethyl esters (Cunnane 2003; Taveira et al. 2009). Ferulic acid was found in the leaves of *R. induratus* (Shafiq et al. 2017), and gallic acid was reported in the roots of *R. dentatus* (Zhu et al. 2006). The names and chemical structures of these compounds are presented in Figure 5.

### 4.3 Anthraquinones

**FIGURE 6:**
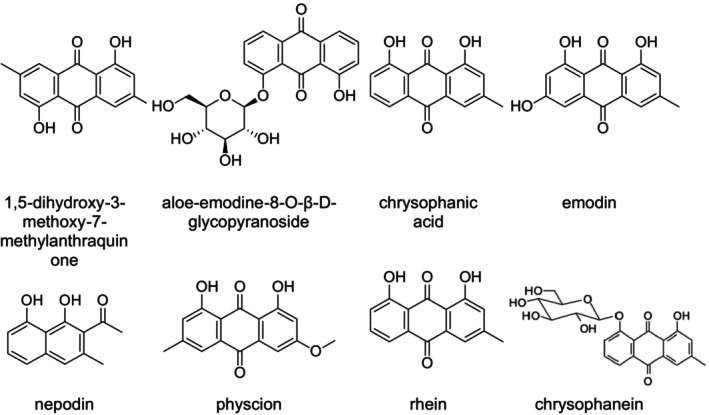
Structures of the reported anthraquinones identified in *Rumex* species, including emodin, chrysophanol, physcion, rhein, and aloe‐emodin derivatives.

Research has indicated that anthraquinones show several biological activities. This is the case with the roots of *R. crispus* , which contain three important anthraquinones: 1,5‐dihydroxy‐3‐methylanthraquinone (ziganein), 1,3,5‐trihydroxy‐6‐hydroxymethylanthraquinone, and 1,5‐dihydroxy‐3‐methoxy‐7‐methylanthraquinone (shiraiarin) (Figure 6). These compounds confirmed the use of the roots of *R. crispus* to purify the blood in skin complaints, and to cure constipation. In addition, these compounds have purgative, anti‐microorganic, and anti‐inflammatory activities (Shafiq et al. 2017). *R. abissinicus* and leaves of *Rumex nepalensis* Spreng. have been indicated to have chrysophanol, which, when given to dysmenorrhea patients, showed antifungal activity (Sharma et al. 2008). The roots of *R. japonicus* , which presented emodine, were reported for curing acute and chronic cutaneous disease (Başkan et al. 2007; Li et al. 2000). Moreover, many *Rumex* species, such as *Rumex acetosa* L., *Rumex acetosella* L., *Rumex confertus* Willd., *R. crispus* , *R. hydrolapathum* Huds., *R. nepalensis* , and *R. obtusifolius* L. have been investigated to identify anthraquinone derivatives such as physcion, physcioni, rhein, and physcion‐8‐*O*‐β‐D‐glucopyranoside (Figure 6). These compounds are significant secondary metabolites in plants and have been identified and isolated through chromatographic methods. All these compounds of these plants can be used in phytotherapy as they deliver a material called *radix lapathi* which has laxative properties (Cos et al. 2002). Nepodin, a key antifungal agent, was extracted from the roots of *R. nepalensis;* although emodin and physcion, isolated from the roots of *R. abyssinicus* , were noted for their antibacterial activity against a broad range of bacteria (Desta 1995). Furthermore, the leaves, roots, and fruits of *R. crispus* and *R. obtusifolius* have been reported to contain sennoside A, a compound widely used as a natural laxative. (Cos et al. 2002). Similarly, 3‐acetyl‐5‐hyroxy‐7‐methoxy‐2‐methyl‐1,4‐naphthaquinone isolated from *R. japonicus* was used as an antimicrobial agent. The anthraquinone derivative aloe‐emodine acetate serves as a precursor for the synthesis of anthracycline antibiotics and exhibits a variety of biological activities, including cathartic, antimicrobial, antiseptic, antibacterial, anti‐mutagenic, and anti‐leukemic effects. The presence of anthraquinone glycosides such as emodine‐6‐*O*‐β‐D‐glucopyranoside, chrysophanein, emodine‐8‐*O*‐β‐D‐glucopyranoside extracted from dried roots of *R. patientia has been investigated*. These last two are used as purgatives, constipation remedies, depuratives, and tonics in Turkish medicine (Demirezer, Kuruüzüm‐Uz, et al. 2001). Additionally, several anthraquinones have been identified in the extract of *R. nepalesis*. These compounds reinforce the traditional medicine system by enhancing intestinal peristalsis, promoting gastrointestinal motility, and exhibiting purgative effects. Aloe‐emodine‐8‐*O*‐β‐D‐glucopyranoside (Figure 6), emodin and nataloin were isolated from *Rumex spinosus* L.

### 4.4 Flavonoids

**FIGURE 7:**
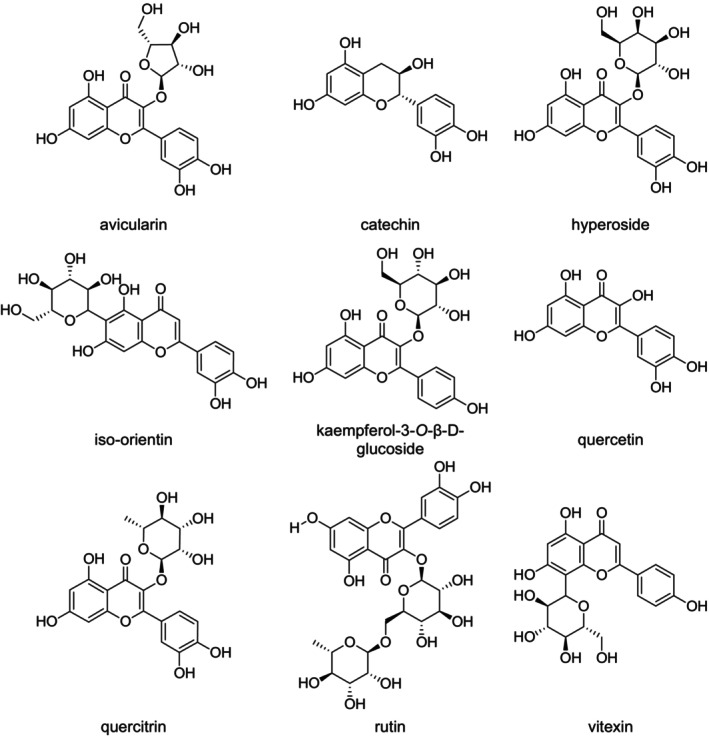
Structure of the reported flavonoids. The flavonoid core with multiple hydroxyl groups enhances antioxidant and anti‐inflammatory activity. Glycosylation (e.g., rutin, hyperoside, quercitrin) improves solubility but may reduce cell permeability. C2–C3 double bonds and 4‐oxo groups (e.g., in quercetin) favor radical scavenging and enzyme inhibition. Substituent positions influence bioactivity potency.

The leaves of various *Rumex* species are traditionally used in the treatment of diverse skin and systemic conditions, which can be attributed to their rich flavonoid content. Compounds such as hyperin, quercetin, quercitrin, iso‐orientin, avicularin, vitexin, and orientin (Figure 7) are present and have been linked to therapeutic applications against hives, ringworm, boils, jaundice, psoriasis, eczema, scabies, itching, acne, and other skin disorders (Shafiq et al. 2017). Studies on *R. japonicus* have identified the presence of quercetin, isoquercitrin, quercitrin, as well as astragalin and catechin (Figure 7) (Spedding et al. 1989). These phytochemicals have shown promising antidiabetic effects and are considered potential agents for the management of diabetes and associated metabolic disorders (Shafiq et al. 2017). Notably, both rutin and quercitrin have demonstrated anticancer properties, particularly against breast cancer. *R. crispus* exhibits strong antioxidant activity attributed to its high flavonoid content. Catechin has been isolated from *R. patientia* , along with halogenated derivatives such as 6‐chlorocatechin, which possess significant free radical‐scavenging capacity and cytotoxic activity (Middleton Jr. et al. 2000). Furthermore, several flavonoids have been detected in *R. nervosus* (Burt 2004; Kerem et al. 2006), which is traditionally used for the treatment of acne, wound healing, typhus, eczema, and rabies, and is also valued for its hypoglycemic and ophthalmic antiseptic properties (Lee et al. 2005). A flavonoid glycoside, querciturone, isolated from *R. aquaticus* (Kim et al. 2010), has been reported to provide protection against gastritis and esophagitis (Clark et al. 2000; Takahashi et al. 2004), although it also exhibits anti‐inflammatory (Hill‐Kapturczak et al. 2001; Zhou et al. 2004), anti‐proliferative (Deng et al. 2004), and anti‐apoptotic effects (Pae et al. 2004). The seeds and leaves of *R. crispus* also display antioxidant and antimicrobial activity, demonstrated by their ability to reduce 2,2‐diphenyl‐1‐picrylhydrazyl (DPPH) radicals and inhibit microbial growth (Warth 1991). In addition, quercetin has been extracted from the roots of *R. dentatus* (Zhu et al. 2006). In *R. vesicarius* , several bioactive compounds have been identified, including orientin (8‐*C*‐glucosyl‐luteolin), vitexin (8‐*C*‐glucosyl‐apigenin), naringin, diosmin (7‐*O*‐rutinosyl‐diosmetin), and catechin (El‐Hawary et al. 2011), all of which contribute to its pharmacological potential.

The diversity of phytochemical constituents across various *Rumex* species is summarized in Table 1.

**TABLE 1:** Phytochemical constituents of *Rumex* species and their biological activities.

Phytochemical class	Compounds	*Rumex* species	Biological activities	References
Fatty acids	Fatty acids — Linoleic acid — Palmitic acid — Stearic acid — Oleic acid Organic acids — Oxalic acid — Citric acid — Tartaric acid Volatile compounds (fatty acid derivatives) — 4‐Hexen‐1‐ol	*R. induratus* *R. abyssinicus* *R. nervosus* *R. dentatus* *R. vesicarius*	Antibacterial Metabolic support Essential lipid source	Shafiq et al. (2017) Fatima et al. (2009) and Mabberley (1997)
Phenolic acids	Benzoic acid, ferulic acid, gallic acid	*R. induratus* *R. dentatus*	Antioxidant Anti‐aging Anticancer Antimicrobial	Cunnane (2003), Taveira et al. (2009) and Zhu et al. (2006)
Anthraquinones	Emodin, chrysophanol, physcion, rhein, nepodin, aloe‐emodin, glycosides (e.g., emodin‐8‐*O*‐β‐D‐glucoside)	*R. crispus* *R. japonicus* *R. abyssinicus* *R. nepalensis* *R. acetosa* *R. obtusifolius* *R. patientia*	Laxative Antimicrobial Antifungal Anti‐inflammatory Anti‐mutagenic	Cos et al. (2002), Sharma et al. (2008), Demirezer, Kuruüzüm‐Uz, et al. (2001) and Desta (1995)
Flavonoids	Quercetin, catechin, rutin, quercitrin, isoquercitrin, hyperoside, orientin, vitexin, naringin, diosmin	*R. japonicus* *R. dentatus* *R. crispus* *R. vesicarius* *R. aquaticus* *R. nervosus* *R. patientia*	Antioxidant Antidiabetic Anticancer Dermatological applications	Shafiq et al. (2017), Spedding et al. (1989), Kim et al. (2010) and El‐Hawary et al. (2011)
Chromones and triterpenoids	Chromones — Rumexone ( *R. dentatus* ) — Rumexol ( *R. dentatus* ) Triterpenoids — Ursolic acid ( *R. japonicus* ) — Betulinic acid ( *R. japonicus* ) — Oleanolic acid ( *R. patientia* ) — β‐Amyrin ( *R. patientia* ) — Hymenocardin ( *R. hymenosepalus* ) — Lupeol ( *R. hymenosepalus* )	*R. dentatus* *R. japonicus* *R. patentia* *R. hymenosepalus*	Anti‐inflammatory hepatoprotective Antitumor	Rao et al. (2011)
Other secondary metabolites	Saponins, alkaloids, tannins	*R. dentatus* *R. japonicus*	Astringent, tonic, antimicrobial, cytotoxic	Shafiq et al. (2017) and Fatima et al. (2009)

## 5 Biological Activities: Underlying Mechanisms of Action

Several biologically active compounds identified in various *Rumex* species have demonstrated hepatoprotective, cytotoxic, antidiabetic, and other pharmacological activities. These findings have encouraged researchers to investigate the potential applications of this genus in medicine and the pharmaceutical industry. The biological activities of *Rumex* species are largely attributed to their diverse chemical constituents, which exhibit anti‐malarial, anti‐inflammatory, and additional therapeutic properties (Getie et al. 2003), anti‐diarrheal, and anti‐viral activities (Cos et al. 2002) and many other activities. Moreover, numerous studies have demonstrated that the *Rumex* genus possesses antioxidant, antiviral, antibacterial, antifungal, immunosuppressive, antitumor, and antimutagenic properties (Lee et al. 2005; Shafiq et al. 2017; Yildirim et al. 2001). The biologically active compounds identified in the *Rumex* genus, such as alkaloids, phenylpropanoids, sulfated compounds, isoprenoids, and peptides, are responsible for different biological activities. In pharmacological studies, extracts or isolated compounds from *Rumex* species have been tested at doses typically ranging from 25 to 500 mg/kg in animal models and from 1 to 1000 μg/mL in in vitro assays, depending on the extract type, species studied, and biological endpoint. In traditional medicine, decoctions or infusions are often administered orally at approximate doses equivalent to 1–5 g/day of dried plant material, though these practices vary by region and preparation method. However, standardization across studies is often lacking, complicating direct comparison (Prakash Mishra et al. 2018).

### 5.1 Antibacterial

The antibacterial activity of *Rumex* species has been investigated using different experimental approaches (such as agar well diffusion and broth dilution methods). Several research groups studied the antibacterial activity of *Rumex* species against Gram‐positive and Gram‐negative bacteria. Wegiera et al. (2011) tested the effects of *Rumex* fruit extracts: *R. acetosa* , *R. acetosella* , *R. confertus* , *R. crispus* , *Rumex hydrolapathum* Huds., and *R. obtusifolius* in inhibiting the growth of seven Gram‐positive ( *Staphylococcus aureus* ATCC 25923, *S. aureus* 209P—ATCC 6538P, *S. epidermidis* ATCC 12228, two *S. aureus* , and two *S. epidermidis* ) and Gram‐negative ( *Escherichia coli* O55, *E. coli* ATCC 3521, *Proteus mirabilis* , *P. vulgaris* , *Klebsiella pneumoniae* , *K. oxytoca* , and *Pseudomonas aeruginosa* ) bacteria. Indeed, the extract of four *Rumex* ( *R. crispus* , *R. hydrolapathum*, *R. obtusifolius* , and *R. confertus* ) showed a minimum inhibitory concentration (MIC) ranging from 62.5 to 125 μg/mL against *Staphylococci*, although for *Escherichia coli* ATCC 3521, *Pseudomonas aeruginosa* , and *Proteus mirabilis* , the MIC ranged between 125 and > 500 μg/mL. In addition, *R. dentatus* root extracts demonstrated antibacterial and antibiofilm activities against clinical isolates of *P. aeruginosa* , with MIC values ranging from 200 to 1000 μg/mL (Khan et al. 2024). The bioactive components gallic acid and emodin were identified, and in silico studies confirmed their interaction with the LasR protein—a key quorum‐sensing transcriptional regulator in *Pseudomonas aeruginosa* that controls the expression of numerous virulence factors. Inhibiting LasR disrupts bacterial communication and biofilm formation, thereby supporting the potential use of *R. dentatus* in treating infections caused by this pathogen (Khan et al. 2024). Similarly, *R. vesicarius* methanolic extracts exhibited antibacterial and antibiofilm properties, notably inhibiting *Bacillus subtilis* and *P. aeruginosa* with MIC values of 250–500 μg/mL (Sulieman et al. 2024). Phytochemical analysis revealed flavonoids, phenolics, tannins, and terpenes as major constituents responsible for these effects (Sulieman et al. 2024). In another study, Hussain et al. (2010) investigated the potential antibacterial effect of methanolic extraction of *R. nepalensis* roots against six bacterial species. The results showed significant activity against *Citrobacter frundii*, *E. coli* , *Enterobacter aerogenes* , and *S. aureus* with MICs of 16, 5.0, 25, and 0.156 μg/mL, respectively. Furthermore, the root, leaves, and stem of *R. nepalensis* have been studied for their antibacterial activity against human pathogenic strains. The results were promoted for leaf extracts. Aqueous leaf extracts of *R. nepalensis* revealed an inhibition zone of 24 mm against *E. coli* (Mungole and Chaturvedi 2011). However, the water extract of stems of *R. nepalensis* exhibited an inhibition zone of 20 mm against *S. aureus* although the water extract of the *R. nepalensis* roots showed an inhibition zone of 19.1 mm against *Pseudomonas* sp. In another study, Yadav et al. (2011) evaluated the effect of methanol, hexane, ethyl acetate, and chloroform of *R. nepalensis* against *S. aureus* , *Salmonella enterica* serovar *Typhimurium*, *E. coli* , and *Klebsiella pneumoniae* . The antibacterial effect of the ethyl acetate fraction revealed a MIC value of 6.3 and 3.1 mg/mL against *S. aureus* and *E. coli* , respectively. Moreover, bioassay‐guided fractionation of *R. abyssinicus* led to the isolation of six antimicrobial anthraquinones, including chrysophanol, emodin, and physcion, with MICs between 8 and 256 μg/mL (Kengne et al. 2021). Naphthalenes such as torachrysone and 2‐methoxy‐stypandrone, isolated from *R. japonicus* , demonstrated bacteriostatic effects against various Gram‐positive and Gram‐negative bacteria (Nishina et al. 1993). Further research on *Rumex* species from the Carpathian Basin revealed strong antibacterial activity, especially in the *n*‐hexane and chloroform fractions of roots from *R. acetosa* , *R. alpinus* , *R. aquaticus* , *R. conglomeratus* , and *R. patientia* , with inhibition zones > 15 mm. Among the compounds tested, naphthalenes exhibited greater antimicrobial capacity (e.g., MIC = 48–57.8 μM for *Moraxella catarrhalis* ) than anthraquinones, flavonoids, stilbenes, and glycerides (Orbán‐Gyapai et al. 2017). Notably, *R. crispus* and *R. sanguineus* showed activity against *Acinetobacter baumannii* , suggesting potential for wound healing applications (MIC = 1.0–4.0 mg/mL) (Aleksic Sabo et al. 2020). In addition, anti‐ *Helicobacter pylori* activity was observed in extracts of *R. acetosa* , where six anthraquinones exhibited MIC values between 3.13 and 25 μM (Kang et al. 2023). These compounds, including emodin and chrysophanol glucosides, also showed urease inhibitory effects, suggesting their potential for managing *H. pylori* ‐related gastric infections (Kang et al. 2023). Furthermore, green synthesis of silver nanoparticles (AgNPs) using *Rumex* sp. leaf extracts showed significant antimicrobial and antibiofilm activity. The AgNPs reduced biofilm formation in *A. baumannii* , *K. pneumoniae* , and *E. coli* , offering a potential nanotechnology‐based solution against resistant pathogens (Akay et al. 2024). Although antibacterial compounds such as anthraquinones and naphthalenes are effective, high concentrations or prolonged use may disrupt normal microbiota or cause mild gastrointestinal irritation.

### 5.2 Antifungal

Several works showed the antifungal effect of different extracts from different plant parts of *Rumex* species. Sharma et al. (2008) studied the effect of root extract of *R. nepalensis* for their antifungal activity against 12 different fungal pathogens. The results demonstrated that the root ethanolic extract of *R. nepalensis* showed a significant active result against the fungal pathogens such as *Fusarium moniliforme*, *Fusarium solani*, *Avicularia versicolor* , *Pythium* sp., *Fusarium semitectum*, *Sporotrichum* sp., *Rhizopus* sp., and *Thermomyces* sp. According to Wegiera et al. (2011), six species of *Rumex* fruit extracts ( *R. acetosella* , *R. acetosa* , *R. crispus* , *R. confertus* , *R. obtusifolius* , and *R. hydrolapathum*) showed antifungal activity using an agar dilution method. The extracts from fruits of *R. crispus* , *R. confertus* , *R. obtusifolius* , and *R. hydrolapathum* inhibited the growth of *Candida* spp. and *Trichophyton mentagrophytes* with MIC values from 250 to 500 μg/mL. Also, the anti‐fungal activity of the methanolic extraction of *R. nepalensis* leaf showed significant activity against *Candida albicans* (Yadav et al. 2011), although *R. nepalensis* root extraction showed higher activity against *A. niger* and moderate activity against *A. solani* , and *A. flavus* (Jain and Parkhe 2018). Extracts from *R. japonicus* leaves exhibited strong in vitro activity against *T. mentagrophytes*, *T. rubrum* , and *C. albicans* (MIC: 1.96–62.5 μg/mL), primarily attributed to anthraquinones such as rhein, emodin, and aloe‐emodin, as well as polyphenols and flavonoids like rutin and quercetin (Xiao et al. 2024). These compounds disrupt fungal spore germination, impair germ tube formation, increase cell membrane permeability, damage biofilms, and downregulate adhesion‐related gene expression. Additional studies confirmed the potent antifungal effects of rhein, emodin, and aloe‐emodin from *R. japonicus* against dermatophytes, including *Microsporum canis*, with MICs ranging from 1.9 to 62.5 μg/mL (Sun et al. 2024). A quality evaluation study of *R. japonicus* highlighted the antifungal activity of emodin‐8‐*O*‐β‐D‐glucopyranoside and ethyl acetate fractions against *C. albicans* (Xiao et al. 2022). Furthermore, ethanolic and aqueous extracts of *R. nervosus* also exhibited antifungal activity (Al‐Garadi et al. 2022). In vitro assays demonstrated complete inhibition of *Aspergillus fumigatus* and significant suppression of *A. niger* mycelial growth. Methanolic leaf and root extracts of *R. nervosus* also showed notable activity against *C. albicans* , as well as several oral bacterial pathogens, supporting its traditional medicinal use (Al‐Farhan et al. 2022). Some antifungal constituents, especially emodin and related anthraquinones, may cause mucosal irritation or hepatotoxic effects at elevated doses or with systemic use.

### 5.3 Antiviral

Several species of the *Rumex* genus have shown notable antiviral properties, particularly against members of the Herpesviridae family. Specifically, 1,4‐naphthoquinones and naphthalene derivatives isolated from *R. aquaticus* exhibited inhibitory effects on the replication of herpes simplex virus type 2 (HSV‐2) in Vero cell cultures. Among these, musizin demonstrated dose‐dependent antiviral activity, resulting in a 2.00 log₁₀ reduction in viral load at a concentration of 6.25 μM, as determined by conventional virus yield reduction assays and quantitative PCR (Rédei et al. 2019). Similarly, an acetone–water extract prepared from the aerial parts of *R. acetosa* , rich in oligomeric and polymeric proanthocyanidins as well as flavonoids, demonstrated strong antiviral effects against herpes simplex virus type 1 (HSV‐1). Using plaque reduction and MTT assays on Vero cells, the extract showed complete inhibition of HSV‐1 at concentrations above 1 μg/mL (IC₅₀ = 0.8 ± 0.04 μg/mL), with minimal cytotoxicity (CC₅₀ = 78.6 ± 12.7 μg/mL) (Gescher et al. 2011). Although antiviral polyphenols are generally well tolerated, excessive intake or long‐term use of concentrated extracts may lead to gastrointestinal upset or interference with micronutrient absorption.

### 5.4 Antioxidant

Gomaa and Saleh (2015) reported significant antioxidant and antiproliferative effects of aqueous extracts from *R. vesicarius* leaves, flowers, and their combination, with the flower extract showing the highest activity in a dose‐dependent manner. Consistently, wild‐grown *R. vesicarius* from the mountains of Hail demonstrated significant antioxidant activity at lower concentrations compared to ascorbic acid, butylated hydroxytoluene (BHT), and β‐carotene, confirming its richness in bioactive phytochemicals and essential minerals (Sulieman et al. 2024). A comprehensive metabolic profiling of *R. vesicarius* identified 60 metabolites, including phenolic acids, flavonoids, terpenes, and organic acids. Molecular docking studies revealed strong binding affinities of predominant metabolites to NADPH oxidase and human peroxiredoxin 5, confirming the stems and roots of *R. vesicarius* as rich sources of antioxidant compounds (Sweilam et al. 2024). Elzaawely and Tawata (2012) evaluated ethyl acetate fractions of leaves and roots of *R. dentatus* , which exhibited strong DPPH radical scavenging activity, with IC_50_ values of 21 and 12 μg/mL, respectively. Similarly, in *R. patientia* , halogenated flavan‐3‐ol‐6‐chlorocatechin and catechin showed potent DPPH scavenging, whereas other compounds such as physcion and emodin derivatives lacked activity (Demirezer, Kuruüzüm, et al. 2001). Methanolic extracts of *Rumex maritimus* L. also demonstrated notable antioxidant potential, with an IC_50_ value of approximately 80 μg/mL compared to 7 μg/mL for ascorbic acid in DPPH assays (Hossain et al. 2015). Antioxidant properties of *R. acetosella* were confirmed through DPPH, ABTS^+^, ${\mathrm{NO}}_{2}^{-}$ radical scavenging, and phosphomolybdate assays (Özenver et al. 2020). Regarding *R. crispus* , antioxidant activity was demonstrated across various assays (DPPH, ABTS+, NO, phosphomolybdate, and SPF), supporting its development as an antioxidant, anti‐aging, and dermatological agent (Uzun and Demirezer 2019). Dichloromethane and ethyl acetate fractions of *R. crispus* roots showed enhanced activity correlated with polyphenol and flavonoid content (Eom et al. 2020). Chrysophanol and physcion extracted from *R. crispus* roots displayed significant xanthine oxidase inhibitory activity, with IC₅₀ values of 36.4 and 45.0 μg/mL, respectively (Minh et al. 2019). In *R. balcanicus Rech.f*., fruits exhibited remarkable antioxidant potential, with IC_50_ values of 4.9 μg/mL (DPPH), 0.8 μg/mL (ABTS), and a FRAP value of 5.9 mmol Fe^2+^/g. These effects were attributed to high contents of miquelianin and procyanidin B1 (Krgović et al. 2025). Furthermore, isolated compounds from *R. hastatus*, including chrysophanol, 1,3,7‐trihydroxy‐6‐methylanthraquinone, przewalskinone B, and *p*‐coumaric acid, demonstrated nitric oxide (NO) radical scavenging activity with IC_50_ values between 0.39 and 0.47 mM (Shafiq et al. 2020). Finally, silver nanoparticles synthesized using *Rumex* sp. leaf extracts exhibited antioxidant activity, benefiting from plant‐derived phytochemicals involved in nanoparticle synthesis (Gam et al. 2024). It is important to note that high doses of antioxidant‐rich extracts, particularly those containing anthraquinones and tannins, can cause gastrointestinal discomfort such as cramping or diarrhea.

### 5.5 Anti‐Inflammatory

Several *Rumex* species have demonstrated anti‐inflammatory properties in both in vitro and in vivo models. In cellular models, methanolic extracts from the roots and stems of *Rumex roseus* L. suppressed the expression of interleukin (IL)‐6 and IL‐8 induced by tumor necrosis factor (TNF)‐α in intestinal epithelial cells, suggesting a regulatory effect on inflammatory pathways (Chelly et al. 2021). For *R. crispus* , an ethyl acetate extract of its roots exhibited marked anti‐inflammatory effects in vitro, including NO inhibition and downregulation of pro‐inflammatory cytokines (Eom et al. 2020). Additionally, *R. crispus* extracts enhanced the suppression of lipopolysaccharide (LPS)‐induced inflammatory responses in RAW 264.7 murine macrophages compared to unprocessed extracts, reducing nitrite levels and inflammatory cytokine expression, likely through modulation of immune signaling pathways (Pan et al. 2020). Furthermore, a formulation composed of processed *R. crispus* and *Cordyceps militaris*, traditionally used in Korean folk medicine, was evaluated for its effects on ovalbumin‐induced allergic rhinitis (AR) in rats. The formulation significantly reduced serum and nasal mucosa levels of immunoglobulin E (IgE), histamine, thymic stromal lymphopoietin, TNF‐α, IL‐1, IL‐4, IL‐5, and IL‐13 (H. Y. Kim et al. 2019). The ethanol extract of *R. japonicus* roots showed therapeutic promise for atopic dermatitis, significantly alleviating skin inflammation in a Balb/c mouse model (Yang et al. 2019). In vitro studies with HaCaT cells demonstrated that the extract reduced mitogen‐activated protein kinase (MAPK) phosphorylation and modulated nuclear factor (NF)‐κB activation in response to TNF‐α stimulation. Additionally, *R. japonicus* methanolic extract protected intestinal epithelial integrity in a dextran sulfate sodium (DSS)‐induced colitis model in C57BL/6N mice (H. R. Yang et al. 2019). For *R. nepalensis* , chloroform and ethyl acetate root extracts significantly reduced ear edema in a 12‐*O*‐tetradecanoylphorbol‐13‐acetate (TPA)‐induced acute inflammation mouse model (Gautam et al. 2010). Mechanistically, these extracts exhibited strong cyclooxygenase (COX)‐1 and COX‐2 inhibition, attributed to isolated anthraquinones (emodin, endocrocin) and naphthalene derivatives (nepodin). Emodin displayed moderate COX‐2 selectivity, although nepodin showed COX‐1 selectivity and potent antioxidant capacity, suggesting a dual mechanism of COX inhibition and radical scavenging in the anti‐inflammatory effect (Gautam et al. 2010). An aqueous extract of *R. patientia* roots exhibited anti‐inflammatory effects in vivo, with the higher tested dose (150 mg/kg) reducing carrageenan‐induced paw edema in rats by 41.7%, a greater inhibition than that observed with the reference drug indomethacin (10 mg/kg, 36.6%) (Süleyman et al. 1999). *Rumex abyssinicus* rhizome extracts demonstrated both wound healing and anti‐inflammatory effects (Mulisa et al. 2015). In vitro, 80% methanol extracts exhibited dose‐dependent anti‐inflammatory activity in the carrageenan‐induced paw edema model. Topical application of extract‐based ointments (5% and 10%) significantly improved wound contraction, epithelialization time, tensile strength, and hydroxyproline content, supporting its traditional use in wound healing (Mulisa et al. 2015). At pharmacological doses, some anti‐inflammatory constituents (e.g., anthraquinones, flavonoids) may induce gastrointestinal irritation or, in rare cases, electrolyte imbalance due to their mild laxative effects.

### 5.6 Hepatoprotective

The antioxidant capacity of *Rumex* species has been associated with their hepatoprotective properties (Table 1). Extracts of *Rumex tingitanus* L. have shown strong radical scavenging activity in vitro, with both the ethyl acetate fraction and the hydroalcoholic extract exhibiting notable antioxidant effects. Additionally, the hydroalcoholic extract demonstrated hepatoprotective activity in a CCl_4_‐induced liver toxicity model, evidenced by reduced lipid peroxidation (MDA levels) and enhanced antioxidant enzyme activities. These effects were further supported by the isolation of 4′‐*p*‐acetylcoumaroyl luteolin, a compound with superior DPPH radical scavenging activity compared to BHT (IC₅₀: 11.7 ± 0.2 μg/mL) (Mhalla et al. 2018). In vitro antioxidant analyses of *R. abyssinicus* rhizome extracts confirmed dose‐dependent radical‐scavenging activity, supporting its traditional use for liver protection (Adamu et al. 2020). Methanolic extracts of *R. vesicarius* (100 and 200 mg/kg body weight) exhibited hepatoprotective activity in CCl_4_‐treated rats (Tukappa et al. 2015). In addition, the methanolic extract of *R. vesicarius* was evaluated for cytotoxicity against HepG2 cell lines, revealing an IC_50_ value of 563.33 ± 0.8 μg/mL, indicating low cytotoxicity and suggesting its safety for further therapeutic applications. The administration of *R. dentatus* at doses of 250 and 500 mg/kg significantly mitigated paracetamol‐induced hepatic damage in mice, as evidenced by reductions in serum ALT, AST, ALP, and bilirubin levels, comparable to the standard hepatoprotective agent silymarin. Histopathological analysis corroborated these findings, although phytochemical screening confirmed the presence of active constituents such as quercetin, kaempferol, and myricetin (Saleem et al. 2014). Ethanol and butanol extracts from roots, leaves, and fruits of *R. vesicarius* significantly reduced hepatic enzyme markers (ALT, AST, ALP) and improved antioxidant status after 4 weeks of treatment at 100 mg/kg, with efficacy comparable to silymarin (50 mg/kg) (El‐Hawary et al. 2011). These effects were attributed to antioxidant mechanisms, membrane stabilization, and antifibrogenic activities (El‐Hawary et al. 2011). *R. abyssinicus* also demonstrated significant hepatoprotection in pre‐ and post‐treatment CCl_4_‐induced liver injury models. Oral administration of its extracts at 125, 250, and 500 mg/kg effectively attenuated the elevation of liver enzymes, with histological analysis revealing preserved hepatic structure (Adamu et al. 2020). Despite hepatoprotective potential, chronic or high‐dose use of Rumex extracts may paradoxically induce liver enzyme elevation or hepatotoxicity, especially in formulations lacking standardization (Figure 7).

**FIGURE 8:**
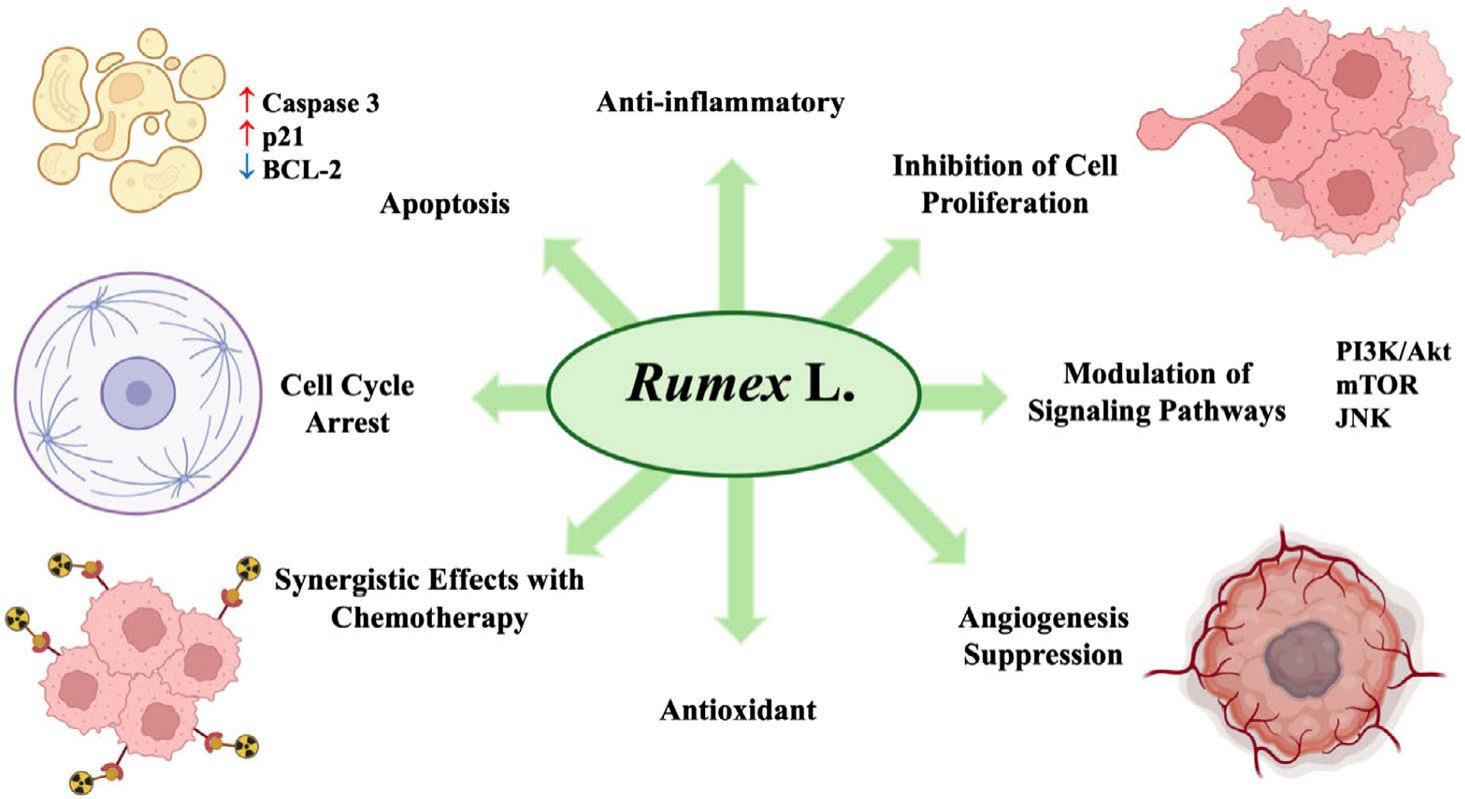
Anticancer mechanisms of *Rumex* L. Bcl‐2, B‐cell lymphoma 2; JNK, c‐Jun N‐terminal kinase; mTOR, mechanistic target of rapamycin; PI3K/Akt, phosphoinositide 3‐kinase/protein kinase B (Akt). Symbols: ↑ increase; ↓ decrease.

### 5.7 Anticancer

MTT assays conducted on HeLa (human cervical carcinoma), A431 (skin epidermoid carcinoma), and MCF7 (human breast adenocarcinoma) cell lines revealed that *R. acetosa* and *Rumex thyrsiflorus* Fingerh. possess the ability to suppress tumor cell proliferation (Lajter et al. 2013). In addition, phenolic extracts from the flowers of *R. acetosa* showed a concentration‐dependent antiproliferative effect on HaCaT cells in vitro. Increasing the extract concentration from 25 to 100 μg/mL progressively reduced cell proliferation (Kucekova et al. 2011).

The methanolic extract of *R. vesicarius* was evaluated for its hepatoprotective properties in vitro, particularly in a model of CCl_4_‐induced liver damage, which was evident at doses of 100 and 200 mg/kg body weight. This plant also demonstrated cytotoxic effects against HepG2 cells, a human liver cancer cell line (Tukappa et al. 2015). Furthermore, dichloromethane extracts obtained from *R. crispus* roots inhibited cell proliferation and triggered apoptosis in HepG2 cells (Eom et al. 2020). 2‐Methoxystypandrone, isolated from *R. japonicus* , displayed antiproliferative activity against Jurkat cells (Qiu et al. 2020). This effect was associated with a reduction in mitochondrial membrane potential and an increase in mitochondrial reactive oxygen species (Qiu et al. 2020). Different parts of the *R. vesicarius* plant—including stems, roots, flowers, and leaves—were tested for cytotoxicity using the MTS assay on several human cancer cell lines, including MCF7, Lovo, Caco‐2 (colon carcinoma), and HepG2 (Farooq et al. 2020). Among these, the stems showed the strongest cytotoxic effect in vitro and did not interfere with zebrafish embryonic development. The IC₅₀ values ranged from 33.45 to 62.56 μg/mL. At a dose of 30 μg/mL, the chloroform extract from the stems significantly suppressed angiogenesis in zebrafish embryos, inhibiting ≥ 70% of intersegmental blood vessels and completely blocking the formation of subintestinal vein vessels, indicating a promising antitumor effect (Farooq et al. 2020).

In triple‐negative breast cancer (TNBC) cell lines (MDA‐MB‐231), *R. vesicarius* extract enhanced the efficacy of sorafenib by downregulating anti‐apoptotic and pro‐survival genes BCl2, mTOR, and JNK, although upregulating the tumor suppressor gene p21 (Ghanem et al. 2023). Molecular docking studies confirmed the high affinity of *R. vesicarius* bioactive compounds for key targets involved in TNBC progression (Ghanem et al. 2023). In *R. dentatus* , a 70% methanolic leaf extract demonstrated weak in vitro inhibition of Ehrlich ascites carcinoma cells, although higher concentrations showed variable anticancer activity, likely due to differential chemical compositions depending on environmental growth conditions (Hawas et al. 2011). Notably, synergy studies combining *R. dentatus* aerial part phenolic aglycones with cisplatin in tongue squamous cell carcinoma (HNO97) cells revealed enhanced inhibition of proliferation, increased apoptosis, cell cycle arrest, and downregulation of autophagy pathways. These effects involved multiple signaling pathways including PI3K‐Akt, microRNAs in cancer, and resistance mechanisms to epidermal growth factor receptor inhibitors (Ragab et al. 2025). Further isolation and characterization of *R. dentatus* extracts identified fractions with potent cytotoxic activity, notably one fraction with an IC₅₀ of 11.29 μg/mL against colon cancer (HCT‐116) cells, which inhibited cell migration and invasion (Khaliq et al. 2023). Studies with *R. obtusifolius* have also shown promising results in combination therapies. The extract combined with 5‐fluorouracil (5‐FU) triggered apoptosis in A549 lung adenocarcinoma cells by modulating the PI3K/Akt pathway and reducing pro‐inflammatory markers such as TNF‐α and COX‐2, enhancing caspase‐3 activity (Ginovyan et al. 2024). Additional investigations combining *R. obtusifolius* extracts with arginase and nitric oxide synthase inhibitors demonstrated immunostimulatory, antiproliferative, antioxidant, anti‐inflammatory, and antiangiogenic effects in breast and colon cancer models (Ginovyan et al. 2023). *Rumex japonicus* root extracts showed protective effects against colitis‐associated colorectal cancer in mice by reducing inflammation, maintaining intestinal barrier integrity, and modulating cytokine profiles, highlighting its anti‐inflammatory and anticancer potential in gastrointestinal cancers (H. Y. Kim et al. 2022). Figure 7 summarizes the main anticancer mechanisms attributed to different *Rumex* L. species. These mechanisms include antiproliferative and pro‐apoptotic effects on various human cancer cell lines (e.g., HeLa, HepG2, MCF7, A549, HCT‐116), inhibition of angiogenesis, and enhancement of chemotherapeutic efficacy (e.g., sorafenib, cisplatin, 5‐FU). Key pathways modulated by *Rumex* extracts or isolated compounds involve PI3K/Akt, mTOR, Bcl‐2, JNK, p21, and microRNAs associated with tumor progression and drug resistance. Overall, the genus *Rumex* exerts its anticancer effects through multi‐targeted actions including apoptosis induction, suppression of angiogenesis, modulation of immune response, and inhibition of tumor cell migration and invasion. Cytotoxic effects of Rumex‐derived compounds may extend to non‐target cells at high concentrations, and long‐term use may carry genotoxic risks without appropriate dose control and safety profiling.

### 5.8 Anti‐Diabetic Activity

Chrysophanol and physcion, isolated from the roots of *R. crispus* , demonstrated inhibitory effects on α‐glucosidase with IC₅₀ values of 20.1 and 18.9 μM, respectively (Minh et al. 2019). The alcoholic extract of *R. acetosella* exhibited a stronger α‐glucosidase inhibitory activity compared to the positive control acarbose (IC₅₀ = 605 μM), with root and aerial parts showing IC₅₀ values of 12.3 μM and less than 10 μM, respectively (Özenver et al. 2020). Furthermore, the methanolic extract from *Rumex lunaria* L. leaves showed significant α‐glucosidase inhibition starting at a concentration of 3 μg/mL (Froldi et al. 2020). Similarly, the methanolic extracts of *R. crispus* flowers and leaves showed potent in vitro α‐glucosidase inhibition with IC₅₀ values of 7.3 and 112.0 μg/mL, respectively, and in vivo antihyperglycemic effects in streptozotocin (STZ)‐induced diabetic rats (Aguila‐Muñoz et al. 2023). The hypoglycemic activity of orally administered ethanol extract from *R. obtusifolius* seeds was assessed in hyperglycemic rabbits (Aghajanyan et al. 2018). Treatment resulted in a 57.3% reduction in fasting glucose levels, enhanced glucose tolerance, and a 1.5‐fold increase in liver glycogen content compared to controls. Additionally, the extract lowered total cholesterol, low‐density lipoprotein cholesterol, and liver enzyme levels while increasing high‐density lipoprotein cholesterol (Aghajanyan et al. 2018). Phenolic compounds extracted from *R. dentatus* were found to improve hyperglycemia by regulating carbohydrate metabolism in the liver, reducing oxidative stress, and upregulating PPARγ expression in diabetic rats (Elsayed et al. 2020). In vivo studies further confirmed that polyphenol‐rich extracts from *R. dentatus* administered orally at doses of 50–200 mg/kg reduce hyperglycemia and insulin resistance while enhancing carbohydrate metabolism in diabetic rats (Y. Yang et al. 2013). The ethanolic extract of *R. vesicarius* leaves exhibited dose‐dependent α‐glucosidase inhibition (up to 60% inhibition at 9 mg/mL) and significantly decreased blood glucose levels in STZ‐induced diabetic rats at doses of 100, 200, and 400 mg/kg (intraperitoneal administration) (Reddy et al. 2017). Histopathological examination confirmed protection of pancreatic β‐cells from STZ‐induced damage. Additionally, thermal treatment of *R. vesicarius* extracts maintained phenolic content and enhanced α‐amylase and α‐glucosidase inhibitory activities (Bouafia et al. 2025). Hydromethanolic root extracts of *R. abyssinicus* showed significant antidiabetic activity in normoglycemic, glucose‐loaded, and STZ‐induced diabetic mouse models, with doses of 100–400 mg/kg reducing blood glucose levels and supporting its traditional use in diabetes treatment (Yrga Adugna et al. 2022). These studies illustrate the principal antidiabetic mechanisms exerted by various *Rumex* species and their bioactive constituents. These include strong in vitro inhibition of α‐glucosidase and α‐amylase enzymes by extracts or isolated anthraquinones (e.g., chrysophanol and physcion), resulting in delayed carbohydrate digestion and postprandial glucose control. In vivo studies demonstrated significant antihyperglycemic effects, improved glucose tolerance, and enhanced hepatic glycogen storage in diabetic animal models. Some species, such as *R. dentatus* and *R. nervosus*, were also shown to reduce oxidative stress and inflammation, thereby contributing to improved insulin sensitivity, protection of pancreatic β‐cells, and prevention of diabetic nephropathy. Figure 8 illustrates the main pharmacological activities of *Rumex* species along with their associated bioactive compounds and molecular targets. Some Rumex extracts may potentiate the effects of antidiabetic medications, increasing the risk of hypoglycemia. Gastrointestinal effects such as nausea and diarrhea have also been reported at higher doses.

**FIGURE 9:**
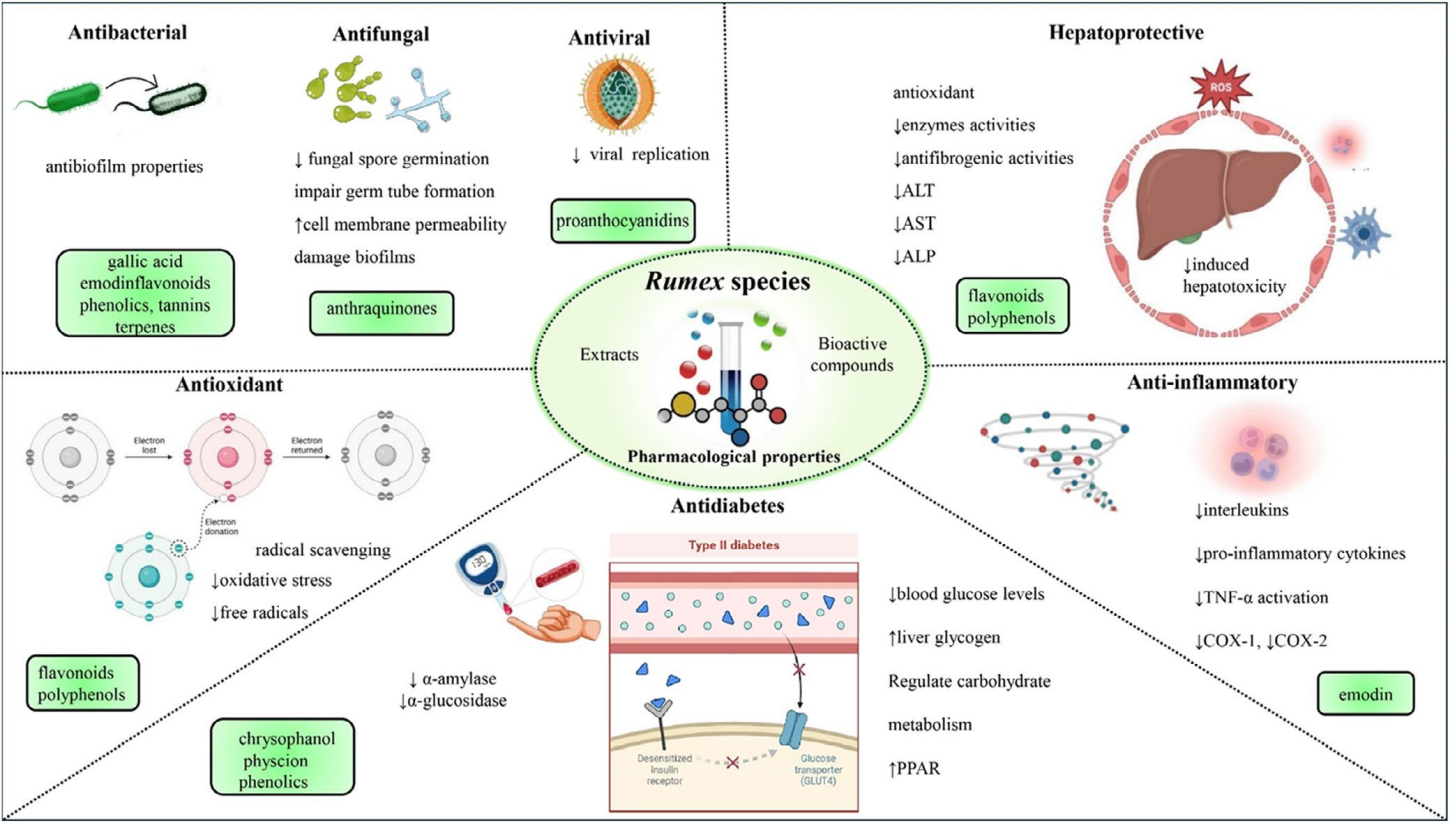
Overview of major pharmacological activities of *Rumex* species and their associated bioactive compounds. Antibacterial activity includes antibiofilm effects mediated by compounds such as gallic acid, emodin, flavonoids, phenolics, tannins, and terpenes. Antifungal effects are primarily attributed to anthraquinones and involve inhibition of fungal germination, increased membrane permeability, and biofilm disruption. Antiviral activity is linked to proanthocyanidins and involves suppression of viral replication. Hepatoprotective effects are mediated by flavonoids and polyphenols, resulting in reduced hepatotoxicity and improved antioxidant enzyme activity. The anti‐inflammatory activity involves emodin and other constituents that downregulate interleukins, TNF‐α, and COX enzymes. Antioxidant effects, driven by flavonoids and polyphenols, include free radical scavenging and reduced oxidative stress. Antidiabetic activity is supported by chrysophanol, physcion, and phenolics through inhibition of α‐amylase and α‐glucosidase, improved glucose uptake, and modulation of carbohydrate metabolism and PPAR expression. ALP, alkaline phosphatase; ALT, alanine aminotransferase; AST, aspartate aminotransferase; COX, cyclooxygenase; PPAR, peroxisome proliferator‐activated receptor; ROS, reactive oxygen species; TNF‐α, tumor necrosis factor‐alpha.

### 5.9 Other Pharmacological Properties

Intravenous administration of the methanolic extract from *R. acetosa* leaves at 50 mg/kg led to a reduction in mean arterial pressure by 27.9% ± 4.6% in normotensive rats, and by 48.4% ± 4.9% in hypertensive rats (Qamar et al. 2018). The antiplatelet properties and cardiovascular protective mechanisms of *R. acetosa* were also investigated. The extract of *R. acetosa* inhibited collagen‐induced platelet aggregation by regulating the phosphorylation of signaling pathways such as MAPK, PI3K/Akt, and Src family kinases, and reduced ATP release in a dose‐dependent manner ranging from 25 to 200 μg/mL (Jeong et al. 2020). Chrysophanol, emodin, and physcion from the aqueous extract of *R. crispus* suppressed osteoclast differentiation induced by RANKL by inhibiting the MAPK/NF‐κB/NFATc1 signaling pathway and increasing inhibitors of NFATc1, thus potentially stimulating osteoblast differentiation via the ERK/Runx2 pathway (Shim et al. 2017). Methanol extracts of *R. nervosus* demonstrated protective effects against STZ‐induced diabetic nephropathy in rats by activating the Nrf2 signaling pathway, reducing oxidative stress, inflammation markers, and apoptosis in renal tissues, thereby preserving kidney function (AlMousa et al. 2022).

Table 2 summarizes the biological effects of various *Rumex* species, detailing the extracts tested, experimental models used, key mechanisms of action, and supporting references.

**TABLE 2:** Biological effects of *Rumex* species.

Species	Extract/compound	Dose/route of administration/concentration	Experimental model	Potential mechanisms	References
*Rumex crispus* , *R. hydrolapathum*, *R. obtusifolius* , *R. confertus*	Methanolic extracts/fruits	MIC: 62.5–125 μg/mL (Staphylococci); 125–500 μg/mL (Gram‐negatives)	In vitro antibacterial assays	Antibacterial activity; flavonoids, phenolics, tannins, terpenes identified	Wegiera et al. (2011)
*Rumex dentatus*	Root extract	MIC: 200–1000 μg/mL	In vitro antibacterial and antibiofilm assays	Antibacterial, antibiofilm activity; gallic acid, emodin bioactives; LasR protein interaction	Khan et al. (2024)
*Rumex vesicarius*	Methanolic extract	MIC: 250–500 μg/mL	In vitro antibacterial and antibiofilm assays	Antibacterial activity; flavonoids, phenolics, tannins, terpenes	(Sulieman et al. 2024)
*Rumex japonicus*	Leaf extract	MIC: 1.96–62.5 μg/mL	In vitro antifungal assays	Antifungal activity; anthraquinones (rhein, emodin), flavonoids (rutin, quercetin); disrupt membrane, biofilm, spore germination	Xiao et al. (2024)
*Rumex nervosus*	Ethanolic, aqueous extracts/leaves, roots	Not specified	In vitro antifungal assays	Antifungal activity; complete inhibition of *Aspergillus fumigatus* ; against *Candida albicans*	Al‐Garadi et al. (2022)
*Rumex aquaticus*	1,4‐Naphthoquinones, naphthalene derivatives	6.25 μM musizin	In vitro antiviral assays (HSV‐2)	Antiviral activity; viral replication inhibition	(Rédei et al. 2019)
*Rumex acetosa*	Acetone‐water extract/aerial parts	IC₅₀ = 0.8 μg/mL (HSV‐1)	In vitro antiviral assays	Antiviral activity; proanthocyanidins, flavonoids; complete HSV‐1 inhibition	Gescher et al. (2011)
*Rumex vesicarius*	Aqueous extract/leaves, flowers	Dose‐dependent	In vitro antioxidant assays	Antioxidant activity; significant radical scavenging; phenolics, flavonoids identified	Gomaa and Saleh (2015)
*Rumex patientia*	Methanolic extract	Not specified	In vitro antioxidant assays	Antioxidant activity; halogenated flavan‐3‐ol and catechin potent DPPH scavengers	Demirezer, Kuruüzüm, et al. (2001)
*Rumex abyssinicus*	80% Methanolic extract/rhizome	125, 250, and 500 mg/kg orally (p.o.)	In vivo—CCl_4_‐induced hepatotoxicity/mice	↓ ALT, AST, ALP at 500 mg/kg; histological preservation of hepatic structure	Adamu et al. (2020)
*Rumex dentatus*	Aqueous methanolic extract/leaves	500 and 250 mg/kg orally (p. o.)	In vivo—paracetamol‐induced hepatotoxicity/mice	↓ ALT, AST, ALP, bilirubin; histopathological protection	Saleem et al. (2014)
*Rumex tingitanus*	Hydroalcoholic extract	250 mg/kg intraperitoneally	In vitro (antioxidant assays); in vivo—CCl_4_‐induced hepatotoxicity rats	↓ MDA; ↑ antioxidant enzymes; compound 4′‐p‐acetylcoumaroyl luteolin showed strong antioxidant activity; no acute toxicity up to 2000 mg/kg orally; antidepressant effect in mice	Mhalla et al. (2018)
*Rumex vesicarius*	Ethanolic extracts/roots, leaves, fruits	100 mg/kg orally (p.o.)	In vivo—CCl_4_‐induced hepatotoxicity/rats	↑ regeneration of parenchymal cells	El‐Hawary et al. (2011)
*Rumex vesicarius*	Methanolic extract	100 and 200 mg/kg orally (p.o.)	In vivo—CCl_4_‐induced hepatotoxicity/rats	↓ liver damage; IC₅₀ (cytotoxicity in HepG2 cells): 563.33 μg/mL → low cytotoxicity, potential therapeutic use	Tukappa et al. (2015)
*Rumex acetosa* , *R. thyrsiflorus*	Phenolic extracts	25–100 μg/mL	In vitro—HeLa, A431, MCF7, HaCaT cells	Antiproliferative activity; concentration‐dependent inhibition	Lajter et al. (2013) and Kucekova et al. (2011)
*Rumex acetosa*	Methanolic extract/leaves	50 mg/kg intravenous	In vivo—normotensive, hypertensive rats	↓ mean arterial pressure; cardiovascular effect	Qamar et al. (2018)
*Rumex acetosa*	Extract	25–200 μg/mL	In vitro antiplatelet assays	Inhibition of platelet aggregation; MAPK, PI3K/Akt, Src regulation	Jeong et al. (2020)
*Rumex crispus*	Aqueous extract	Not specified	In vitro osteoclastogenesis assay	Suppressed osteoclast differentiation; MAPK/NF‐κB/NFATc1 inhibition	Shim et al. (2017)
*Rumex nervosus*	Methanolic extract	Not specified	In vivo—STZ‐induced diabetic nephropathy rats	↓ oxidative stress; ↓ inflammation; ↓ apoptosis; Nrf2 activation	AlMousa et al. (2022)
*Rumex abyssinicus*	Hydromethanolic extract/root	100–400 mg/kg orally	In vivo—diabetic mouse models	↓ blood glucose; improved glucose tolerance	Yrga Adugna et al. (2022)
*Rumex dentatus*	Phenolic compounds	50–200 mg/kg orally	In vivo—diabetic rats	↓ hyperglycemia; ↓ oxidative stress; ↑ PPARγ	Elsayed et al. (2020)
*Rumex obtusifolius*	Ethanol extract/seeds	Not specified	In vivo—hyperglycemic rabbits	↓ fasting glucose by 57.3%; improved lipid profile	Aghajanyan et al. (2018)
*Rumex lunaria*	Methanolic extract/leaves	3 μM	In vitro α‐glucosidase assay	Significant α‐glucosidase inhibition	Froldi et al. (2020)
*Rumex crispus*	Methanolic extract/flowers, leaves	7.3 and 112.0 μg/mL (IC₅₀)	In vitro α‐glucosidase assay; in vivo diabetic rats	Potent α‐glucosidase inhibition; antihyperglycemic effect	Aguila‐Muñoz et al. (2023)

Abbreviations: ALP, alkaline phosphatase; ALT, alanine aminotransferase; AST, aspartate aminotransferase; COX, cyclooxygenase; IC₅₀, half‐maximal inhibitory concentration; MAPK, mitogen‐activated protein kinase; MDA, malondialdehyde; MIC, minimum inhibitory concentration; NF‐κB, nuclear factor kappa B; NFATc1, nuclear factor of activated T‐cells 1; PPARγ, peroxisome proliferator‐activated receptor gamma; STZ, streptozotocin.

## 6 Clinical Studies

Species of the genus *Rumex* have been studied in diverse clinical contexts. *R. acetosa* is included as one of the five herbal components in the fixed combination extract BNO 1016 (Sinupret, Bionorica SE, Germany), developed for the treatment of sinusitis (Jund et al. 2012). Preclinical studies have demonstrated antimicrobial, antiviral, anti‐inflammatory, and secretolytic properties of the formulation. A Phase IIb/III clinical trial identified 160 mg, administered three times daily, as the optimal dose. This dosage was found to be effective and well tolerated over a 15‐day treatment period in patients with acute viral rhinosinusitis.

In dermatological applications, a randomized, double‐blind, placebo‐controlled trial evaluated the efficacy and safety of a 3% *Rumex occidentalis* S.Watson cream compared to a 4% hydroquinone (HQ) cream in the treatment of melasma among Filipino patients (Mendoza et al. 2014). Over 8 weeks, all treatment groups showed reductions in pigmentation as measured by the Melasma Area Severity Index (MASI) and mexameter readings. By week 8, the *R. occidentalis* group exhibited comparable or greater improvements than the HQ group, with both treatments rated similarly effective by investigators and participants. The study concluded that *R. occidentalis* cream is a safe and effective alternative to HQ for skin lightening in melasma management. In the context of oral health, a randomized, placebo‐controlled pilot study assessed the effects of a mouth rinse containing 0.8% proanthocyanidin‐enriched *R. acetosa* extract in systemically healthy carriers of *Porphyromonas gingivalis* (Selbach et al. 2023). Although no significant intergroup differences in microbiological or cytological parameters were observed, intragroup analysis showed a significant reduction in the Sulcular Bleeding Index and Approximal Plaque Index at days 7 and 14 in the *Rumex* group. The mouth rinse was well tolerated, with no serious adverse events reported.

## 7 Toxicity and Side Effects

*Rumex* plants contain notable amounts of oxalic acid, which can cause health issues when ingested in large quantities. High dietary oxalate intake is associated with secondary hyperoxaluria and an increased risk of calcium oxalate kidney stones. Oxalates also reduce the absorption of calcium and other minerals by forming insoluble complexes, decreasing their bioavailability. The risk of poisoning can be reduced by avoiding consumption of the cooking water, where oxalate concentration may be higher. Due to these risks, *Rumex* consumption should be avoided by individuals with kidney stones, gout, arthritis, rheumatism, or hyperacidity. The estimated lethal dose of oxalic acid in adults ranges from 15 to 30 g, with lower doses (< 5 g) sometimes causing fatal outcomes (A. Vasas et al. 2015). A summary of other clinically reported toxicities associated with *Rumex* species is provided in Table 3.

**TABLE 3:** Reported human toxicities and adverse effects associated with *Rumex* species.

Rumex species	Toxic components	Reported clinical effects	References
*R. crispus* (yellow dock, curly dock)	Oxalic acid; anthraquinone glycosides (emodin)	Fatal oxalate poisoning (hypocalcemia, metabolic acidosis, hepatic/renal failure) after large ingestion; also causes nausea, vomiting, diarrhea, electrolyte imbalances (hypokalemia), and rare immune thrombocytopenia from tea use	Reig et al. (1990)
*R. acetosella* (sheep sorrel)	Oxalic acid; anthraquinones (emodin, chrysophanol)	Diarrhea, cramping, and gastroenteritis with overuse; renal impairment and hepatotoxicity in large doses (oxalate nephropathy, elevated liver enzymes); contraindicated in kidney stone sufferers	Vasas et al. (2015)
*R. acetosa* (common sorrel)	Oxalic acid (abundant in leaves)	Acute kidney injury (oxalate crystal nephropathy) reported in a child after excessive sorrel ingestion; risk of hypocalcemia and multi‐organ oxalosis; Llarge amounts can cause nausea and mucosal irritation	Selçuk et al. (2015)
*R. dentatus* (toothed dock)	Anthraquinones Tannins	Severe gastrointestinal distress (dysentery, bloody diarrhea, gastric pain) noted in ethnomedicine reports if used improperly as a purgative Symptoms of colic and dehydration can occur with high doses	Qazi et al. (2022)
*R. vesicarius* (bladder dock)	Oxalic acid; anthraquinones (less in leaves)	No acute human poisonings recorded; high oxalate content suggests risk of kidney stone formation if overconsumed Mild laxative effect; large portions may upset stomach or cause hyperacidity	El‐Hawary et al. (2011)
*R. obtusifolius* (broadleaf dock)	Polyphenols oxalates	Contact dermatitis from sap (skin rash upon handling leaves); infrequent ingestion can cause oropharyngeal irritation Not recommended as food due to potential skin and GI irritation	Vasas et al. (2015)

## 8 Limitations and Clinical Gaps

Although the genus *Rumex* exhibits a wide array of promising pharmacological activities, several critical limitations hinder its progression toward clinical application. The current evidence base is dominated by preclinical studies; human clinical trials evaluating safety, efficacy, and dosing remain virtually absent. A further constraint is the considerable heterogeneity in study design, with inconsistent methodologies for extract preparation, characterization, and phytochemical analysis, making cross‐study comparisons difficult. In addition, most studies do not systematically account for variables such as species differences, plant part selection, harvest timing, and environmental influences, all of which can affect phytochemical composition. Pharmacokinetic and bioavailability data for key bioactive compounds are scarce, and most reports do not address issues of solubility, absorption, metabolism, or tissue distribution. Toxicological profiles for many *Rumex* species, especially under chronic administration, remain incomplete, leaving gaps in our understanding of long‐term safety. Furthermore, herb–drug interaction potential has not been adequately explored, which is an important consideration in populations with polypharmacy. Addressing these limitations will be essential to reliably characterize the therapeutic potential of *Rumex* species and guide future research toward clinically meaningful outcomes.

## 9 Conclusion and Future Prospects

*Rumex* species have long been integral to traditional medicine systems across diverse cultures. Notably, several phytoconstituents, including anthraquinones, flavonoids, tannins, stilbenes, and naphthalenes, have been identified in *Rumex* species, with a broad spectrum of biological effects such as antioxidant, antimicrobial, antidiabetic, hepatoprotective, and cytotoxic activities. Nevertheless, important gaps remain. First, the phytochemical profiles of many Rumex species remain poorly studied in relation to plant developmental stages. Environmental factors can also significantly influence the yield and composition of secondary metabolites. Second, although in vitro and in vivo studies provide encouraging data on biological activities, there is a notable lack of well‐designed clinical trials and standardized extracts. Third, current knowledge about the pharmacokinetics and bioavailability of key *Rumex* phytoconstituents is limited, which hinders the translation of promising preclinical findings into effective therapeutic applications. Given that compounds such as emodin, chrysophanol, flavonoids and tannins exhibit poor bioavailability in their native form, future research should prioritize formulation strategies that improve solubility, absorption, and stability. However, such enhancements must be approached cautiously, as increased bioavailability could also elevate the risk of toxicity, particularly for compounds with narrow therapeutic windows. Toxicological evaluations are scarce and scattered, particularly for lesser‐studied species and chronic administration models. Therefore, detailed toxicokinetic investigations and long‐term toxicity assessments are needed. In conclusion, *Rumex* species represent a rich but underutilized reservoir of bioactive compounds with substantial pharmacological potential. Future research should aim to (i) explore interspecies phytochemical variability in relation to phenology and habitat, (ii) develop standardized bioactive extracts, (iii) conduct pharmacokinetic and clinical studies and (iv) rigorously evaluate toxicological safety. These efforts would pave the way toward the rational development of *Rumex*‐based therapeutics and functional products, fully integrating traditional knowledge with modern biomedical approaches.

## Author Contributions

**Aya Khouchlaa:** writing – original draft, writing – review and editing, visualization, validation, methodology, investigation, data curation. **William N. Setzer:** writing – original draft, investigation, data curation, validation, methodology, visualization, writing – review and editing. **Daniela Calina:** writing – review and editing, writing – original draft, investigation, validation, visualization, methodology, project administration, supervision, data curation. **Shahira Mohamed Ezzat:** investigation, writing – original draft, writing – review and editing, visualization, validation, methodology, supervision, data curation. **Miquel Martorell:** writing – original draft, writing – review and editing, visualization, validation, methodology, investigation, data curation, supervision. **Javad Sharifi‐Rad:** writing – original draft, writing – review and editing, project administration, supervision, data curation, methodology, validation, visualization, investigation, conceptualization. **Seham Salaheldin Elhawary:** writing – original draft, writing – review and editing, visualization, validation, methodology, investigation, data curation. **Balakyz Yeskaliyeva:** writing – original draft, writing – review and editing, visualization, validation, methodology, investigation, data curation. **Farid Noshey Kirollos:** writing – original draft, writing – review and editing, visualization, validation, methodology, investigation, data curation.

## Funding

The authors have nothing to report.

## Conflicts of Interest

The authors declare no conflicts of interest.

## Data Availability

The authors have nothing to report.
